# Effects of hypothermia combined with neural stem cell transplantation on recovery of neurological function in rats with spinal cord injury

**DOI:** 10.3892/mmr.2014.2905

**Published:** 2014-11-10

**Authors:** DONG WANG, JIANJUN ZHANG

**Affiliations:** 1 Department of Neurosurgery, The Fourth Center Clinical College of Tianjin Medical University, Tianjin 300140, P.R. China; 2 Department of Neurosurgery, General Hospital of Tianjin Medical University, Tianjin 300052, P.R. China

**Keywords:** spinal cord injury, neural stem cells, transplantation, hypothermia, rat

## Abstract

The microenvironment of the injured spinal cord is hypothesized to be involved in driving the differentiation and survival of engrafted neural stem cells (NSCs). Hypothermia is known to improve the microenvironment of the injured spinal cord in a number of ways. To investigate the effect of NSC transplantation in combination with hypothermia on the recovery of rat spinal cord injury, 60 Sprague-Dawley female rats were used to establish a spinal cord hemisection model. They were divided randomly into three groups: A, spinal cord injury group; B, NSC transplantation group; and C, NSC transplantation + hypothermia group. At 1, 2, 4, 6 and 8 weeks post-injury, the motor function of all animals was evaluated using the Basso, Beattie and Besnaham locomotor scoring system and the inclined plane test. At 4 weeks post-transplantation, histological analysis and immunocytochemistry were performed. At 8 weeks post-transplantation, horseradish peroxidase nerve tracing and transmission electron microscopy were conducted to observe axonal regeneration. The outcome of hind limb motor function recovery in group C significantly surpassed that in group B at 4 weeks post-injury (P<0.05). Recovery was also observed in group A, but to a lesser degree. For the pathological sections no neural axonal were observed in group A. A few axon-like structures were observed in group B and more in group C. Horseradish peroxidase-labeled neurofibers and bromodeoxyuridine-positive cells were observed in the spinal cords of group C. Fewer of these cells were found in group B and fewer still in group A. The differences among the three groups were significant (P<0.05). Using transmission electron microscopy, newly formed nerve fibers and myelinated nerve fibers were observed in the central transverse plane in groups B and C, although these nerve fibers were not evident in group A. In conclusion, NSC transplantation promoted the recovery of hind limb function in rats, and combination treatment with hypothermia produced synergistic effects.

## Introduction

With industrialization and the development of more advanced forms of transportation, the incidence of spinal cord injury (SCI) has increased. SCI is a significant cause of morbidity and mortality (1). Spinal cord injuries comprise damage that results in complete or partial loss of sensation and/or motor control, and can therefore have a marked effect on quality of life (2–4). Current treatment options include surgery, medicine, such as Ganglioside and Oxiracetam and physiotherapy, but no therapy is yet available to completely restore function.

Increasing evidence has shown that brain tissue-derived neural stem cells (NSCs) have the potential for self-proliferation and multilineage differentiation under certain conditions. NSCs are able to differentiate into a variety of cells within the nervous system, indicating that they may be used for the treatment of nerve injury (5). However, NSC transplantation alone is not sufficient for spinal cord repair, since the majority of the cells implanted into the spinal cord have been shown to differentiate into a phenotype that is restricted to glial lineages, and which rarely survive. The microenvironment of the injured spinal cord is hypothesized to be important in inducing the differentiation and survival of grafted NSCs (6,7). In recent years, hypothermia (33–35°C) has become an increasing focus of attention in research into the treatment of SCI and brain injury, due to its neuroprotective effects against secondary injury (8).

A large number of clinical studies have shown that hypothermia effectively reduces secondary brain and SCI injury, and also protects the central nervous system from injury. The beneficial effects of hypothermia include reducing oxygen consumption, decreasing free radical generation, delaying the release of damaged neurotransmitters, reducing inflammation, lowering metabolic demand and preventing the formation of cytotoxic edema. Even a temperature reduction of 1–2°C has been demonstrated to be protective against secondary neurological injury at the cellular level in any organ or tissue (9–13).

In the present study, it was hypothesized that hypothermia improves the differentiation and survival of engrafted NSCs via its effects on the microenvironment of the injured spinal cord. To investigate this hypothesis, the microenvironment was modified by hypothermia, during transplantation of NSCs in a model of SCI. The aim of this study was to investigate the effect of NSC transplantation in combination with hypothermia on the recovery of SCI in rats.

## Materials and methods

### Experimental animals and reagents

This study was approved by the Scientific Review Committee and the Institutional Review board of Tianjin Medical University (Tianjin, China) and all experimental procedures adhered to the Helsinki Declaration. One 1-month-old Sprague Dawley (SD) rat and 60 healthy female SD rats (200–250 g) were obtained from the Chinese Academy of Medical Sciences Animal Laboratory (Beijing, China). L-Dulbecco’s modified Eagle’s medium (L-DMEM) was obtained from Gibco Life Technologies (Carlsbad, CA, USA). Fetal bovine serum was obtained from GE Healthcare Life Sciences (Logan, UT, USA). 0.01 mol/l phosphate-buffered saline (PBS) powder (pH 7.2) was obtained from Fuzhou Maxim Biotech Inc. (Fuzhou, China). Glutamate was obtained from Sigma-Aldrich (St. Louis, MO, USA). Trypsin was obtained from Gibco Life Technologies. EDTA was obtained from Tianjin Chemical Reagent No. 1 Plant (Tianjin, China). Horseradish peroxidase (HRP) was obtained from Santa Cruz Biotechnology, Inc. (Dallas, TX, USA). 5-bromo-deoxyuridine (Brdu) was obtained from Takara Biotechnology, Inc. (Dalian, China). Monoclonal mouse-anti-BrdU antibodies were obtained from Boehringer Manheim (Ingelhemin am Rhein, Germany). Horse anti-mouse IgG polyclonal antibodies conjugated to biotin were obtained from Vector Laboratories, Inc. (Burlingame, CA, USA).

The one-month-old male SD rats were used to collect BMSCs (n=5 rats per group). All the rats were sacrificed via decapitation.

### Rat bone marrow stem cell (BMSC) cultivation

The one-month-old SD rats (irrespective of gender) were sacrificed via decapitaton and disinfected using 75% alcohol for ~10 min. Bilateral removal of tibias and femurs was conducted under sterile conditions. Bone ends were removed, washed in L-DMEM (1 ml) or stored in it or both, and prepared in single-cell suspension at a density of 3×10^4^ cells/ml. Cells were inoculated into 100-ml culture flasks and placed into an incubation box at 37°C, with 5% CO_2_ saturated humidity. The culture liquid was replenished 24 h later and thereafter renewed every three days. Nonadherent cells were removed, and adherent cells were expanded until they reached confluence and processed through sequential passages. The majority of contaminating hematopoietic stem cells were lost after the first passage, and following the second passage, cultures contained a morphologically homogenous cell population, designated BMSCs. This was confirmed by fluorescence-activated cell-sorting analysis, which demonstrated a lack of expression of typical hematopoietic cell surface markers, including CD45, CD34 and CD14, and positivity for CD71, CD105 and CD44. Mesenchymal stem cells CD44, CD90, and CD105 were positively expressed, while CD34 and CD45 were negatively expressed. Cells between passages three and six were used for subsequent experiments. They were labeled using a medium containing BrdU (Takara Biotechnology, Dalian, China).

### Establishment of animal models

A total of 60 female SD rats (200–250 g) were fed standard animal feed (GB14924.2–2001) in the laboratory for 2 weeks and then anesthetized with an intraperitoneal injection of 2.5% ketamine 20 mg/kg (Hainan Kai-Pharmaceutical Co., Ltd., Hainan, China). In the prone position, rats were fixed on the operating table in order to enable preparation of skin specimens, which were then thoroughly disinfected. T9 spinous processes were identified and 2–3 cm of skin and subcutaneous tissue overlying this area were incised along the posterior median line. Paraspinal muscles were stripped and the T8–T9 spinous processes and lamina were exposed. Using rat forceps, T8 and T9 spinous processes and lamina were removed, exposing the dura mater. The right side of the spinal cord was then cut. Paralysis of the right hind limb was considered to indicate a successful model of SCI. Wounds were rinsed with penicillin (Hainan Kai-Pharmaceutical Co., Ltd., Hainan, China) and saline, and then sutured. Subsequently, the passage of urine was encouraged twice per day, morning and evening, by squeezing the rats’ bladders, until the micturition reflex was restored.

### Animal grouping and mild hypothermia treatment

An HP-V26 temperature meter (Beijing Zhongxiyuanda Technology Co., Ltd., Beijing China) was used for continuous monitoring of rat rectal temperature. The 45 rats in which a model of acute SCI had been established, were randomly divided into three groups: Group A, SCI control group; group B, single BMSC transplantation group, in which rats were placed on the operating table at room temperature with rectal temperature maintained at (37±0.5)°C and at 6 h, a 1 ml BMSC (1×10^10^/l) suspension was administered intravenously through the tail using a 1-ml syringe; and group C, mild hypothermia and BMSC transplantation group, in which rats were placed on an ice blanker machine (Zhuhai Heima Medical Instrument Co., Ltd.), with rectal temperature maintained at (34±0.5)°C, and at 6 h, a 1 ml BMSC (1×10^10^/l) suspension was administered intravenously into the tail using a 1-ml syringe. Then animals were fed in separate cages.

### Functional recovery evaluation

Following treatment, two forms of test were used to assess functional recovery. Each test was observed by two independent investigators.

### Basso, Beattie and Bresnahan (BBB) score

The open-field locomotion test assesses movement, weight support and coordination. It was scored using the standardized BBB locomotor scoring system (12). BBB scores range from 0 (flaccid paralysis) to 21 (normal gait). Rats were acclimated to the test environment (90 cm diameter plastic wading pool; 4 cm height) prior to testing. The test was performed at 1, 2, 4, 6 and 8 weeks post-SCI. The mean BBB score was calculated for each group.

### Inclined plate test

An inclined plate surface was covered with a 6-mm-thick rubber pad and rats were placed in a direction of body axis perpendicular to the longitudinal axis of the inclined plate. The incline angle was gradually increased and rats were required to stay in the inclined plate for at least 5 sec to record the maximum angle achieved. The angle of incline was measured three times in each rat, and the average value was obtained. The three groups were measured at 1, 2, 4, 6 and 8 weeks post-SCI. The mean values for each group at each time point were obtained.

### Histological analysis

Four weeks following SCI, two rats were randomly selected from each group for histological analysis. Dissected spinal cord tissues were post-fixed for 3 h in 4% paraformaldehyde, soaked overnight in 10% and then 30% sucrose, and cut into 15-mm sagittal and parasagittal sections using a cryostat. Hematoxylin and eosin staining, and 1% cresyl violet staining were conducted for general histological examination.

### Immunocytochemistry

Four weeks following SCI, two rats were randomly selected from each group for immunocytochemistry analysis using BrdU. This process required the pre-treatment of tissue sections to denature DNA. All staining was conducted on free-floating 40-μm sections. A monoclonal mouse-anti-BrdU antibody (1:100 dilution) was used in combination with avidinbiotin complex and a horse-anti-IgG-antibody conjugated with biotin (1:167 dilution). Ten fields from each slice were randomly selected and viewed under a high-power microscope (x200) (Metallurgical Microscope; Shanghai Optical Instrument Production Company, Shanghai, China). The mean number of theBrdU-positive cells in each field of vision was calculated for each sample.

### HRP retrograde neural tracing

Eight weeks following SCI, two rats were randomly selected from each group for HRP retrograde neural tracing. Following surgery, the spinal cord was exposed at T12 and 1 μl aqueous suspension of 30% HRP (RZ>3.0, which represented the enzyme purity) was injected 1 mm bilaterally to the spinal dorsal vein. Following injection, the wound was closed and tissue samples of the animals were maintained for 36 h prior to being perfused by with buffer and then fixed with 1% paraformaldehyde and 1.25% glutaraldehyde. Spinal cords were removed and stored in 20% sucrose in 0.1 M PBS at 4°C overnight. The spinal cord was dissected and ten fields from each slice were randomly selected in which to calculate the HRP-labeled neurofibers under a high-power microscope (x200). The mean was calculated for each group.

### Electron microscopy (EM)

Eight weeks following SCI, two rats from each group were randomly selected using a simple random sampling method on pre-labeled rats. They were sacrificed and perfused intracardially with saline, followed by 2% glutaraldehyde and 4% paraformaldehyde in 0.1 M sodium cacodylate buffer (pH 7.4). Immediately following perfusion, the spinal cords were removed and post-fixed in the same medium (comprising a mixture of the primary and secondary antibody) overnight at 4°C. The spinal cord segment at the injury epicenter was sliced into 1-mm sections, post-fixed for 2 h in 1% OsO_4_ in 0.1 M cacodylate buffer, dehydrated in graded ethanol solutions and embedded in Epon-812 (Hyde Venture (Beijing) Biotech Co., Ltd.). Plastic sections (1 μm) were cut and stained with 1% toluidine blue prior to examination with a Nikon Eclipse TE300 microscope (Tokyo, Japan) equipped with a Spot RT Color CCD camera (Basler, Genmany). For EM, blocks were trimmed and sections were cut at 100 nm, mounted on copper grids, stained with uranyl acetate and lead citrate, and viewed with a JEOL Jem 1200 EX transmission electron microscope (JEOL, Tokyo, Japan).

### Statistical analysis

Data are expressed as the mean ± standard deviation in this randomized control trial design. Analysis of variance was performed using SPSS 16.0 statistical software (SPSS Inc., Chicago, IL, USA). Two sample comparison was conducted using Dunnett’s t-test. P<0.05 was considered to indicate a statistically significant difference. All analyses were performed with SPSS statistical software (version 16.0).

## Results

### Morphology of NSCs

**Figure 1:**
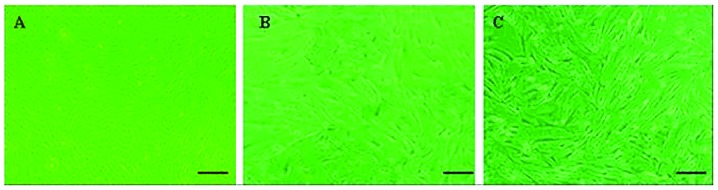
Morphology of neural stem cells. (A) Rat mesenchymal stem cells were cultured for 2 days. Adherent cells extended and became spindle-shaped. (B) Rat mesenchymal stem cells were cultured for 7 days and grown around a clone. (C) The 3rd passage of mesenchymal stem cells fused together and became arranged in a bunched or radiating shape. Scale bar, 50 μm.

The number of bone marrow stromal cells and colonies were significantly increased on the fifth day of culture. Cells at passages 1–3 proliferated actively and the majority of cells adhered to the monolayer, with various morphological forms, including spindle-shaped, oval-shaped, flat-shaped, triangular and irregular cell bodies. Cells exhibited strong refraction and possessed >2 processes, some of which connected to each other, showing nucleus and nucleolus. When the cells were confluent, they were observed to grow in a parallel or spiral manner (Fig. 1).

### BBB scores

**Figure 2:**
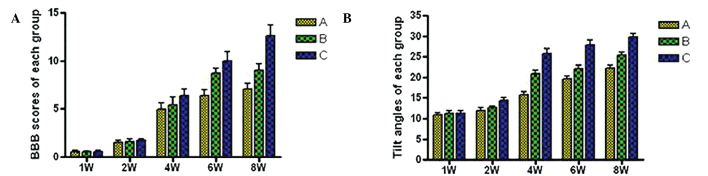
BBB scores and incline plate test. (A) BBB scores and (B) Tilt angles of each group at 1, 2, 4, 6 and 8 weeks following spinal cord injury. BBB, Basso, Beattie and Bresnaham locomotor scoring system. Significance levels are as shown in Tables I and II.

Following SCI, rats manifested full monoplegia with no activity of the right hind limb or tail, and urinary dysfunction but no dysfunction of defecation. The retraction to the puncture, including the manifestation of the movement from contraction to stretch of hind legs, began to emerge at 1 week post-injury. Hind limb movement occurred at 2 weeks post-injury and became increasingly evident at 4 weeks. Hind limbs demonstrated coordination of activities at 6 weeks and urinary function was partially restored, although there was still residual urine in the bladder. The three groups exhibited the same changes following injury. BBB scores in groups B and C were higher than those in group A. At 4 weeks post-injury, group C scores were significantly higher that those from group A (P<0.01) and group B (P<0.05; Table I, Fig. 2A).

### Incline plate test

At 4 weeks post-injury, scores from group C were significantly higher than those from group A (25.8±1.1 compared with 15.7±0.8°, P<0.05) and from group B (25.8±1.1 compared with 20.9±0.9°, P<0.05). Scores from group B were also significantly higher than those from group A (20.9±0.9 compared with 15.7±0.8, P<0.05). At 6 weeks post-injury, there remained significant differences between groups A and C (P<0.01) and between groups B and C (P<0.05). These results suggest that mild hypothermia in combination with NSC transplantation is superior to NSC transplantation alone in terms of functional motor recovery following SCI (Table II, Fig. 2B).

### Histological analysis and immunocytochemistry

**Figure 3:**
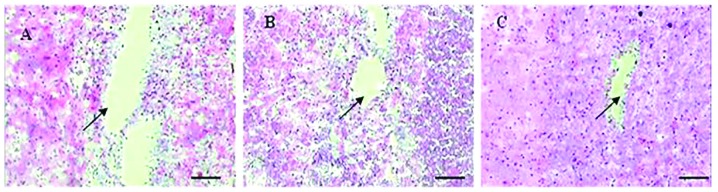
Histological analysis using H&E staining. (A) Four weeks post-injury, in group A at the affected site of the damaged spinal cord exhibited a clear cavity formation. (B) In group B, astrocytes congregated at the edge of the affected site and formed scars at the junction of complete spinal cord and damaged spinal cord. The tissue cavity was smaller than that in group A but larger than that in group C. (C) In group C, astrocytes underwent a reactive hypertrophy, and congregated and formed scars at the edge of the affected site, resulting in the cavity disappearing. Arrows show Syringomyelia and scar healing. Scale bar, 100 μm.

**Figure 4:**
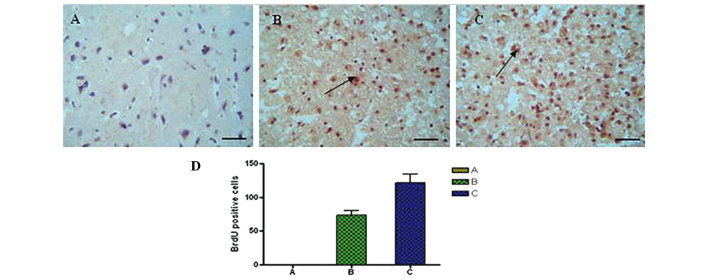
Immunohistochemical staining showing the number of BrdU-positive cells in the SCI lesion tissues in rats in (A) Group A, (B) group B and (C) group C. (D) Graph displaying quantity of BrdU-positive cells in each group. By analysis of variance and Dunnett’s *t*-test comparison, the number of BrdU-positive cells in group C was found to be increased compared with group B (P<0.05) and group A (P<0.01). Arrows show BrdU-positive cells. Scale bar, 50 μm. BrDU, bromodeoxyuridine; SCI, spinal cord injury.

At 4 weeks following injury, spinal cord tissue damage, scarless healing and structural disorder were visible at the affected site in group A, with a clear cavity formation (Fig. 3A). In group B, astrocytes aggregated at the edge of the affected site and formed scars at the junction between the intact and damaged sections of the spinal cord. The cavity in group B was smaller than in group A but larger than in group C (Fig. 3B). In group C, astrocytes underwent reactive hypertrophy, aggregated and formed scars at the edge of the affected site. A number of cells were spindle-shaped, with a dense network between processes. The cavities were not visible in this group (Fig. 3C). Immunohistochemical staining showed the number of BrdU-positive cells in tissues from the SCI lesions (Fig. 4). Using analysis of variance and Dunnett’s t-test, the number of BrdU-positive cells in group C (Fig. 4C) was found to be significantly increased compared with group B (Fig. 4B; P<0.05), and compared with group A (Fig. 4A; P<0.01), at 4 weeks post-injury.

### HRP retrograde nerve tracing

**Figure 5:**
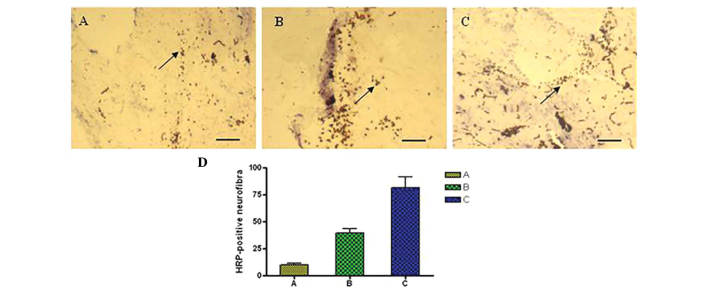
HRP retrograde nerve tracing. (A) In group A, few HRP-positive granule-labeled nerve fibers were observed. (B) In group B, fewer HRP-positive nerve fibers were observed than in group C, but more than group A. (C) In group C, a large quantity of HRP-positive granule-labeled nerve fibers were observed. (D) Graph displaying the number of HRP-positive cells in each group. The number of HRP-positive nerve fiber bundles in rat SCI tissues exhibited significant differences among the three groups at 8 weeks post-injury (P<0.01). Arrows show HRP-positive granule-labeled nerve fibers. Scale bar, 50 μm. HRP, horseradish peroxidase; SCI, spinal cord injury.

DAB color reaction was performed according to manufacturer’s instructions (Shanghai ZiYi Co., Ltd.). A central area of deeply-stained tissue and a surrounding area of less strongly-stained tissue was observed at the injection site. In group A, rats were injected with HRP through the lumbar intumescentia. Two days after HRP injection, the HRP had been transported in a retrograde direction for groups A and B. In segments T8 and above few HRP-positively labeled nerve fibers were observed (Fig. 5A). In group B, HRP-positive nerve fibers were also observed, and there were fewer fibers in group C, although more than in group A (Fig. 5B). Group C exhibited a large quantity of HRP-positive granule-labeled nerve fibers in the spinal cord (Fig. 5C). The number of HRP-positive nerve fiber bundles in rat SCI tissues from each group is shown in Fig. 5D). There were significant differences among the three groups at 8 weeks post-injury (P<0.01).

### Transmission electron microscopy

**Figure 6:**
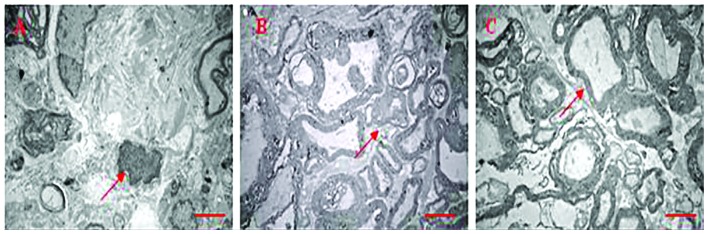
Transmission electron microscopy. (A) A glial scar and a small number of myelinated nerve fibers are shown in group A. (B) The number of myelinated nerve fibers and non-myelinated nerve fibers at the injury site in group B was greater than that in group A but less than that in group C. (C) Numerous myelinated and non-myelinated nerve fibers were observed in group C, with more axons and intact myelin. Arrows show a glial scar and a small number of myelinated nerve fibers. Scale bar, 0.1 μm.

Transmission electron microscopy results showed the glial scar and a small number of myelinated nerve fibers in group A, along with macrophage phagocytosis and degeneration, and necrotic myelinated nerve fibers (Fig. 6A). A large number of myelinated and non-myelinated nerve fibers were observed in group C, which had more axons and intact myelin as compared with the other groups (Fig. 6B). The number of myelinated and non-myelinated nerve fibers at the injury site in group B was greater than that in group A and less than that in group C (Fig. 6C).

## Discussion

Central nervous system regeneration is a complex area of theoretical research and clinical practice in the fields of neuroscience and medicine, and an effective treatment for damage to the nervous system has not yet been developed. Central nervous system injury is primarily a result of trauma, including cerebral cortex impairment or loss of function and paralysis as a result of SCI (14–16). Recently, with the development of stem cell research, NSC transplantation for the treatment of neurological diseases has become a significant focus in medical research (17–19). NSCs have a number of superior qualities as compared to neurons, such as ease of harvesting, well-developed methods for cell separation, culture, amplification and exogenous gene transfection, and the feasibility of autologous implantation following *in vitro* amplification or genetic engineering modification, without encountering ethical issues or immune rejection. NSC transplantation has been shown to effectively treat nervous system injury in a previous study (20). Its mechanisms of action are diverse. NSCs exhibit a high expansion potential, genetic stability and a stable phenotype. They are easily collected and transported, and are compatible with different delivery methods and formulations (21). In addition, NSCs have two other important characteristics: They are able to migrate to sites of tissue injury and they have strong immunosuppressive properties that can be exploited for successful autologous or heterologous transplantation without the requirement for pharmacological immunosuppression (22,23). NSCs are capable of differentiating into neurons and astrocytes *in vitro* and *in vivo* (24). Recently, NSC injection has shown promising results in the treatment of amyotrophic lateral sclerosis in humans (25). They have been shown to improve neurological deficits and promote the development of neuronal networks with functional synaptic transmission, when transplanted into animal models of neurological disorders, such as nerve dysfunction (26). NSCs have been observed to migrate to injured tissues and to mediate functional recovery following brain, spinal cord and peripheral nerve lesions (27).

In recent years, mild hypothermia (33–35°C) has received increasing attention in the treatment of central nervous system injury. A previous clinical study showed that mild hypothermia effectively reduces secondary nerve injury and protects against severe traumatic brain injury (28). The mechanisms underlying this protective effect may include reducing the release of excitatory amino acids, inhibiting calcium influx, regulating calmodulin kinase II and protein kinase C activity, inhibiting the inflammatory response following cerebral ischemia, suppressing edema formation, reducing the oxygen metabolic rate, diminishing the production of free radicals, and inhibiting necrosis and neuronal apoptosis induced by mitochondrial release of cytochrome *c* (29–31). In the present study, the effect of mild hypothermia combined with NSC transplantation on SCI in rats was investigated. The results showed that NSC transplantation combined with mild hypothermia was superior to NSC transplantation alone, in the treatment of SCI in rats, as evaluated by changes in histology and functional recovery.

The synergistic effect of hypothermia and NSC transplantation may be due to the fact that hypothermia improves the microenvironment of the injured spinal cord. An important mechanism underlying the neuroprotective effects of hypothermia is a reduction or delay in metabolic consumption during the period of stress experienced by the injured spinal cord (32–36). The hemodynamic consequences of cooling the spinal cord are important, as reductions in blood flow to critical levels caused by profound cooling may have adverse effects on tissue preservation and thus on functional outcome (37–39). It is clear that the neurotransmitter response in various types of SCI models may be temperature-dependent, but that attenuating other injury cascade may be more important in subserving the beneficial effects of hypothermia (40–43). Alterations in blood-brain barrier permeability following ischemia and trauma are an important vascular consequence that leads to the passage of water, blood-borne exogenous substances and potential neurotoxic agents across the vascular system and into the brain parenchyma. Microvascular perturbations including blood-brain barrier permeability, the formation of vasogenic edema and the extravasation of circulating inflammatory cells may adversely affect injury outcome. The effects of hypothermia on the vasculature comprise an important mechanism contributing to the beneficial effects of hypothermia (44–47). There are also pronounced changes in calcium-dependent intracellular signaling pathways following SCI. The neuronal cytoskeleton is highly vulnerable to injury, resulting in beading of dendrites and degeneration of axons, changes that are reversed by hypothermia. This effect is likely to be mediated by the inhibition of calpain activity, a calcium-dependent protease (48–52). Attenuation of inflammation is one of the major mechanisms by which hypothermia leads to beneficial effects in SCI. The inflammatory response following SCI is known to be significantly attenuated by hypothermia. In addition to attenuating the disruption of the blood-brain barrier and the extravasation of infiltrating inflammatory cells and neurotoxic substances, the endogenous inflammatory response induced by SCI is also reduced by hypothermia (53–55). Evidence for apoptotic cell death has been demonstrated in various models of SCI. Although neuronal necrosis is commonly observed in injury models, evidence for apoptotic cell death in CNS injury has also been documented using various histochemical and molecular techniques. As with necrosis, apoptotic cell death appears to be sensitive to post-injury hypothermic treatment strategies. Using terminal deoxynucleotidetransferase-mediated dUTP-biotin nick end labeling staining, DNA fragmentation has been found to be reduced by hypothermia in SCI (56–59). Recent studies have utilized various genetic markers in order to evaluate the effects of temperature on molecular events associated with SCI. Families of genes associated with inflammation, apoptosis and other cell signaling cascades are known to be reduced or elevated when brain temperature is lowered. The ability of post-injury temperature to affect the acute and delayed genetic responses to injury is important, as these genes may be important in determining the proteomic response that results in secondary injury (60–63).

In conclusion, NSC transplantation in combination with mild hypothermia may promote the survival, proliferation, differentiation and migration of the transplanted cells at the injury site, as well as promoting the restoration of nerve function in rats with SCI. This therapy provides novel strategies and methods for the clinical treatment of SCI.

## Figures and Tables

**Table I:** BBB scores of each group at different time points following spinal cord injury.

	BBB score				
	
Group	1 week	2 weeks	4 weeks	6 weeks	8 weeks
A	0.54±0.15	1.49±0.28	4.97±0.68^a^	6.39±0.59^a^	7.03±0.61^a^
B	0.53±0.12	1.62±0.23	5.42±0.83^b^	8.68±0.52^b^	9.04±0.62^b^
C	0.54±0.11	1.73±0.14	6.39±0.67^a,b^	9.98±0.64^a,b^	12.62±0.73^a,b^

BBB scores for each group at different time points following spinal cord injury. Three groups exhibited the same change following injury. BBB scores in groups B and C were higher than those in group A. Data are presented as the mean ± standard deviation. At four weeks post-injury, group C compared with group B, P<0.05; group C compared with group A, ^a^P<0.01 (Fig. 2A). BBB, Basso, Beattie and Bresnahan locomotor scoring system.

**Table II:** Tilt angles of each group at different time points following spinal cord injury.

	Tilt angle (°)				
	
Group	1 week	2 weeks	4 weeks	6 weeks	8 weeks
A	10.8±0.5	12.0±0.6	15.7±0.8^a^	19.6±0.8^a^	22.2±0.8^a^
B	11.2±0.7	12.6±0.4	20.9±0.9^b^	22.1±0.9^b^	25.4±0.7^b^
C	11.3±0.6	14.4±0.7	25.8±1.1^a,b^	27.8±1.2^a,b^	29.7±1.0^a,b^

Tilt angles of each group at different time points following spinal cord injury. Data are presented as the mean ± standard deviation. At 4 weeks post-injury, group B compared with group A, P<0.05; group C compared with group A. P<0.05; and group C compared with group B, P<0.05. At 6 weeks post-injury, there were significant differences between groups A and C (P<0.01) and between groups B and C (P<0.05; Fig. 2B).
